# Eating Disorders in the Context of Gastrointestinal Diseases—A Narrative Review

**DOI:** 10.3390/nu18183045

**Published:** 2026-09-17

**Authors:** Agnieszka Kopystecka, Beata Kasztelan-Szczerbinska, Halina Cichoz-Lach

**Affiliations:** 1 Doctoral School of the Medical University of Lublin, 20-093 Lublin, Poland; d.agnieszkakopystecka@umlub.edu.pl; 2 Department of Gastroenterology and Hepatology with Endoscopy Unit, Medical University of Lublin, 8 Jaczewski, 20-954 Lublin, Poland; halina.lach@umlub.edu.pl

**Keywords:** eating disorders, disorders of gut–brain interaction, anorexia nervosa, avoidant/restrictive food intake disorder, bulimia nervosa, disorders of gut–brain interaction, feeding and eating disorders

## Abstract

**Background/Objectives:** The clinical overlap between gastrointestinal (GI) conditions and psychiatric disorders represents a challenge in clinical practice. Eating disorders (EDs), including anorexia nervosa, bulimia nervosa, binge eating disorder (BED), and avoidant/restrictive food intake disorder (ARFID), may co-occur with gastrointestinal (GI) diseases and complicate their management. Orthorexia nervosa is a proposed and emerging construct characterized by pathological preoccupation with healthy eating and rigid dietary practices. This narrative review aims to summarize current evidence regarding eating disorder symptomatology, ARFID screening and diagnosis, orthorexic symptomatology, pathophysiological mechanisms, and diagnostic challenges across gastrointestinal diseases, with particular attention to inflammatory bowel disease (IBD), disorders of gut–brain interaction (DGBIs), celiac disease, eosinophilic esophagitis, upper gastrointestinal motility disorders, and the potential role of the gut microbiota. **Methods:** The review was structured in accordance with the SANRA guidelines. A structured literature search was conducted primarily in PubMed, with Scopus and Google Scholar used as supplementary sources for identifying additional relevant publications through citation tracking and examination of reference networks, for articles published from 2011 through early 2026. **Results:** The available literature indicates an association between GI disorders and disordered eating, with potentially bidirectional relationships involving symptom burden, dietary restriction, psychological factors, and gut–brain signaling. Chronic GI symptoms often necessitate dietary modifications, which can inadvertently trigger or mask pathological restrictive eating behaviors. Diagnosing ARFID and orthorexia in these cohorts is particularly challenging due to overlapping somatic symptoms. Primary EDs are also associated with gastrointestinal symptoms and alterations in the gut microbiome; however, human evidence remains predominantly observational, and the temporal and causal relationships between dysbiosis, nutritional restriction, inflammation, and gastrointestinal dysfunction remain uncertain. **Conclusions:** The convergence of EDs and GI conditions requires heightened clinical vigilance. Distinguishing between necessary, symptom-driven dietary restrictions and pathological eating behaviors is crucial. Implementing an integrated, multidisciplinary care model-involving gastroenterologists, psychiatrists, and dietitians-is essential to improve diagnostic accuracy and patient outcomes.

## 1. Introduction

Eating disorders (EDs) are complex conditions characterized by persistent disturbances in eating behaviors with a worldwide weighted-mean lifetime prevalence estimated at 8.4% in women and 2.2% in men, and a point prevalence that has risen markedly over the past two decades [1]. Beyond their psychological impact, EDs are associated with significant medical morbidity, frequent hospitalizations, and a high mortality rate, making them among the most lethal mental disorders [2]. Classic entities such as anorexia nervosa (AN), bulimia nervosa (BN), and binge eating disorder (BED) are well-documented for their severe gastrointestinal (GI) complications, ranging from motility disorders to structural damage [3,4,5,6]. In recent years, the diagnostic landscape has expanded to include avoidant/restrictive food intake disorder (ARFID) and the proposed entity of orthorexia nervosa. Unlike traditional EDs, ARFID is driven by sensory sensitivities, low interest in food, or fear of aversive consequences—such as abdominal pain—rather than body image disturbances. Similarly, orthorexia involves a pathological obsession with “healthy” eating, which can lead to extreme dietary restrictions and malnutrition [1,2,6,7,8,9,10]. Studies using screening instruments have reported relatively high proportions of patients with GI diseases who screen positive for ARFID or exhibit ARFID-related risk, although these estimates should not be interpreted as confirmed diagnostic prevalence because screening instruments may not reliably distinguish pathological food avoidance from symptom-driven dietary restriction [11]. Emerging evidence suggests a profound, bidirectional link between EDs and gastrointestinal conditions, including inflammatory bowel disease (IBD), disorders of gut–brain interaction (DGBIs), and celiac disease [12,13]. Chronic GI symptoms may precede or be associated with disordered eating, while primary EDs are associated with alterations in gastrointestinal function and gut microbial composition. However, the directionality and clinical significance of these associations remain incompletely understood [14,15]. Currently, there is a notable absence of formal guidelines from major gastroenterological societies such as AGA, ECCO, BSG, ESPGHAN, or ESPEN regarding screening for eating disorders within emergency departments and gastroenterology clinics. This significant gap in research highlights an urgent need for action, as patients with disorders of gut–brain interaction (DGBI) frequently present with underlying disordered eating patterns that remain undiagnosed. Recent studies, including work by Atkins et al. (2023) [16] and Murray, H.B. et al. (2022) [17], underscore the necessity of evaluating the psychological and nutritional context of dietary interventions to prevent the exacerbation of ARFID symptoms. Given the growing clinical overlap between these conditions, understanding the relationship between various GI diseases and the spectrum of eating disorders is of critical importance. There is a pressing need to help clinicians distinguish between necessary, symptom-driven dietary modifications and pathological behaviors. Therefore, the concrete aim of this narrative review is to summarize current knowledge regarding the prevalence, pathophysiology, and diagnostic challenges of various EDs in patients with IBD, DGBIs, celiac disease, eosinophilic esophagitis, upper gastrointestinal motility disorders, and gut microbiota alterations [14,15,16,17].

## 2. Materials and Methods

This narrative review was conducted in accordance with the Scale for the Assessment of Narrative Review Articles (SANRA) guidelines. Between 20 December 2025 and 15 January 2026, a structured literature search was conducted primarily in PubMed, with Scopus and Google Scholar used as supplementary sources for the identification of additional relevant publications through citation tracking and examination of reference networks of key articles. The search strategy used Boolean operators ‘AND’ and ‘OR’ and incorporated terms representing the principal eating disorder entities and gastrointestinal conditions relevant to the objectives of the review. The searches were conducted iteratively using topic-specific combinations of these terms, including combinations addressing eating disorders and inflammatory bowel disease, intestinal and gastrointestinal diseases, ARFID and bowel diseases, ARFID screening instruments, and eating disorders and the gut microbiome. These included: (‘eating disorders’ OR ‘anorexia nervosa’ OR ‘bulimia nervosa’ OR ‘binge eating disorder’ OR ‘avoidant/restrictive food intake disorder’ OR ‘ARFID’ OR ‘orthorexia nervosa’) AND (‘inflammatory bowel disease’ OR ‘IBD’ OR ‘Crohn’s disease’ OR ‘ulcerative colitis’ OR ‘intestinal diseases’ OR ‘digestive system diseases’ OR ‘gastrointestinal diseases’ OR ‘disorders of gut–brain interaction’ OR ‘DGBI’ OR ‘irritable bowel syndrome’ OR IBS’ OR ‘celiac disease’ OR ‘bowel diseases’ OR ‘gut microbiome’ OR ‘microbiota’ OR ‘dysbiosis’). Additional targeted searches addressed the relationship between ARFID and screening instruments, including combinations of ‘ARFID’ and ‘NIAS’, as well as the relationship between eating disorders and the gut microbiome.

The search was restricted to English-language, peer-reviewed articles published between January 2011 and January 2026 (a lower bound chosen to capture research preceding DSM-5 ARFID and Rome IV criteria). Selected pre-2011 publications were nevertheless included when they provided seminal diagnostic definitions, original development or validation of relevant assessment instruments, or essential foundational evidence that could not be adequately represented by later publications. The final literature search and citation-tracking process was completed on 15 January 2026. Records were screened based on title, abstract, and full-text eligibility. Exclusion criteria comprised: (1) studies lacking direct clinical or pathophysiological overlap between EDs and GI diseases; (2) animal/in vitro models (except for foundational microbiota mechanistic studies); (3) case reports with n < 5; (4) non-peer-reviewed commentaries or conference abstracts lacking full quantitative data; and (5) publications in languages other than English. Literature identification and initial relevance assessment were performed by one reviewer (A.K.). Bibliographic records were managed using reference management software, and duplicate records were identified and removed before the final selection of publications for synthesis. Because the review was conducted as a narrative review with an iterative literature-identification process rather than as a prospectively registered systematic review, no formal inter-reviewer disagreement procedure was applicable. Additional relevant publications could be identified during subsequent examination of reference lists and citation networks of key studies.

A PRISMA-style sequence of literature identification and selection was conceptually followed, comprising identification of potentially relevant records, removal of duplicate records using bibliographic reference management software, screening of titles and abstracts, assessment of relevant full-text publications, and selection of studies for narrative synthesis. Because an exact database-level screening log was not prospectively maintained, reconstructed record counts were not used as quantitative PRISMA flow estimates.

Terminology regarding ARFID assessment was standardized throughout the review. The term ‘positive ARFID screen’ refers exclusively to a screening instrument result above the predefined threshold. ‘ARFID risk’ refers to a screening-defined likelihood or symptom profile suggestive of ARFID and does not constitute a clinical diagnosis. The term ‘clinically diagnosed ARFID’ is reserved for studies in which ARFID was established using DSM-based clinical assessment or a structured diagnostic interview. Accordingly, screening positivity estimates are not considered equivalent to diagnostic prevalence. Where 95% confidence intervals were not reported in the original publications, they were estimated from the reported numerator and denominator using the Wilson score method. These calculated intervals were used solely for descriptive presentation in Table 1 and do not represent re-analysis of the original study data.

**Figure 1:**
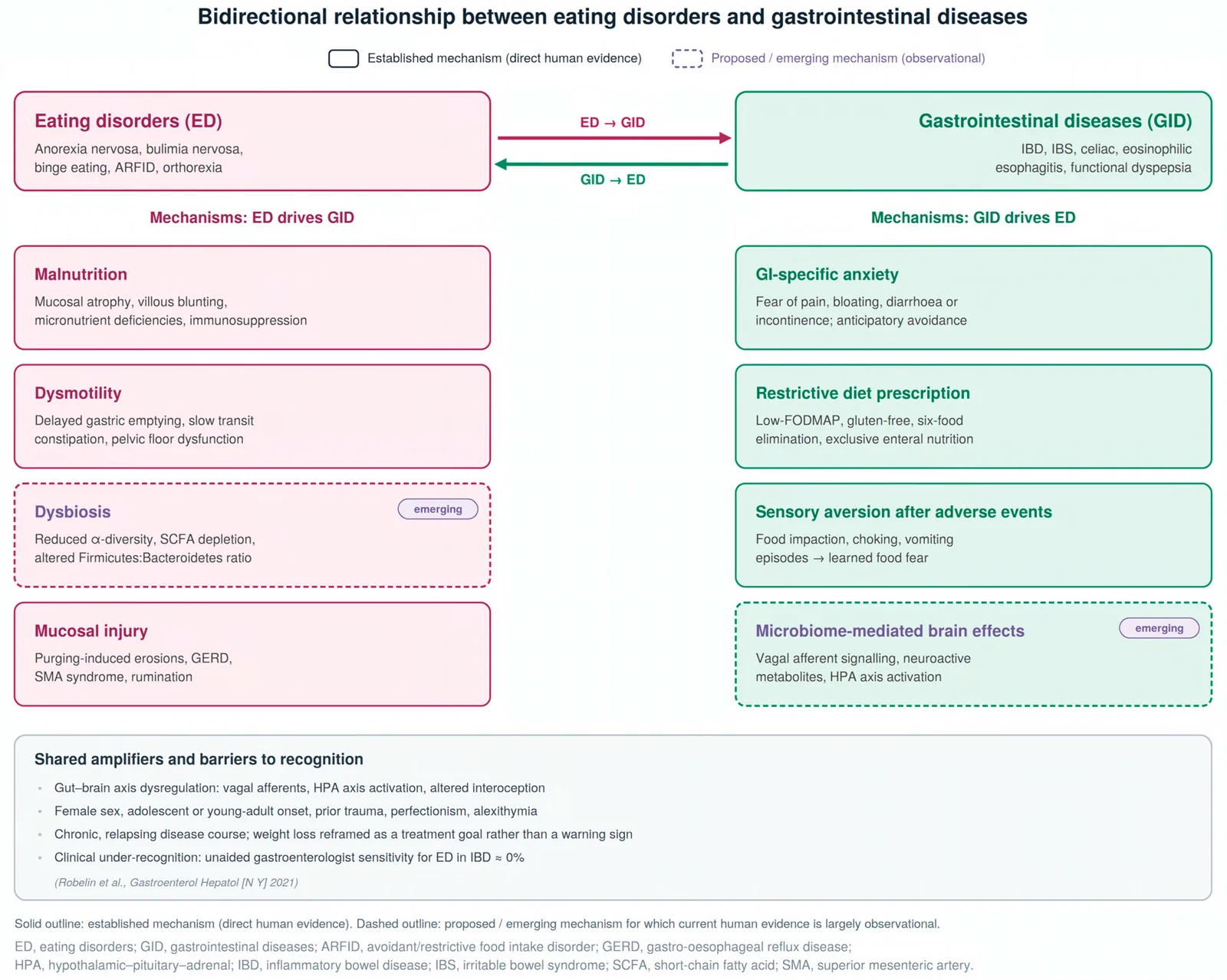
Bidirectional relationship between eating disorders and gastrointestinal diseases. The left column lists four mechanisms by which eating disorders may contribute to gastrointestinal disease: malnutrition, dysmotility, dysbiosis, and mucosal injury. The right column lists four mechanisms operating in the opposite direction, by which gastrointestinal disease may contribute to disordered eating: gastrointestinal-symptom-specific anxiety, restrictive therapeutic diet prescription, sensory aversion after adverse luminal events, and microbiome-mediated effects on brain circuits. The shared amplifiers shown below operate in both directions and contribute to the chronic, self-perpetuating nature of the comorbidity, alongside well-documented clinical under-recognition of eating disorders in gastroenterological practice. Consistent with the evidence hierarchy shown in the figure, established consequences are drawn with solid outlines, whereas dysbiosis and microbiome-mediated effects on brain circuits are shown as proposed pathways linking eating disorders and gastrointestinal disease (dashed outlines) that are not yet supported by established causal evidence. ARFID, avoidant/restrictive food intake disorder; GERD, gastro-oesophageal reflux disease; HPA, hypothalamic–pituitary–adrenal; IBD, inflammatory bowel disease; IBS, irritable bowel syndrome; SCFA, short-chain fatty acid; SMA, superior mesenteric artery [18].

Quality Assessment and SANRA Self-Assessment: Although formal systematic risk-of-bias scoring was not applied due to study design heterogeneity, quality was informally appraised by evaluating sample sizes, diagnostic validity (e.g., use of validated instruments like NIAS, PARDI, or Rome IV vs. non-validated self-reports), and statistical control for confounders (e.g., disease activity, psychiatric comorbidities). The manuscript fulfills the six SANRA criteria: (1) justification of the review’s importance (Section 1); (2) statement of concrete aims (Section 1); (3) detailed description of the literature search (Section 2); (4) comprehensive referencing; (5) rigorous scientific reasoning regarding gut–brain pathways; and (6) presentation of structured data via dedicated synthesis tables (Table 1 and Table 2). In preparing this manuscript, the authors used Claude Opus 4.7 (Anthropic) and Gamma (https://gamma-app.ai) to assist in creating the schematic Figure 1 as stated in the Acknowledgments.

## 3. Clinical Characterization of Classic and Newly Defined Eating Disorders

### 3.1. Anorexia Nervosa, Bulimia Nervosa, and Binge Eating Disorder

Anorexia nervosa (AN), bulimia nervosa (BN), and binge eating disorder (BED) remain the most frequently diagnosed eating disorders. Lifetime prevalence estimates among women may reach up to 4% for anorexia nervosa, approximately 2% for bulimia nervosa, and approximately 2% for binge eating disorder; rates are consistently lower in men and in countries with lower levels of economic development [47,48]. Although treatments are constantly improving [48], eating disorders still contribute to over 7000 deaths per year, making them the most lethal of all mental disorders [49], and the financial burden of treating eating disorders on both the healthcare system and society is very high [2].

Anorexia nervosa (AN) is an eating disorder with a very high mortality rate, affecting women in 95% of cases [31]. It is characterized by a strong fear of weight gain and a significant reduction in BMI, maintained through restricting food intake, excessive physical activity, and/or the use of purging methods. The etiology of anorexia nervosa is multifactorial [31,50] and most likely involves the interaction of genetic predisposition and various environmental factors; significant changes in the composition and functioning of the intestinal microbiota have also been described in patients with this disorder [31]. This intricate biological synergy underscores the contemporary paradigm of AN as a complex metabo-psychiatric pathology, wherein psychiatric symptomatology is fundamentally intertwined with systemic metabolic, endocrine, and neurobiological dysregulation. Consequently, a comprehensive characterization of AN necessitates transitioning from solitary psychopathological evaluations to an integrated biochemical framework that pairs peripheral somatic signals—including gut–brain peptides and microbiota-derived metabolites—with central indices of neuroplasticity and functional neural connectivity [51].

Bulimia nervosa (BN) is a psychiatric disorder characterized by recurrent episodes of binge eating followed by compensatory behaviors aimed at avoiding weight gain [5].

Binge eating disorder (BED) is defined as a clinical entity characterized by recurrent episodes of compulsive eating of objectively large amounts of food within a short period of time, accompanied by loss of control over eating, experiencing marked discomfort, and intense emotional distress. BED is classified as a distinct psychiatric diagnosis in the DSM-5 [52], and in many cases requires the implementation of targeted psychotherapeutic interventions and/or pharmacological treatment [53].

### 3.2. Avoidant/Restrictive Food Intake Disorder (ARFID)

An example of a newly described eating disorder is avoidant or restricted feeding disorder (ARFID). This expanded the previous category associated primarily with feeding disorders occurring in infants and young children. ARFID can be diagnosed in individuals of any age if their restrictive or avoidant eating patterns lead to inadequate energy and/or nutrient intake and, consequently, cause at least one of the following: significant weight loss, severe nutritional deficiencies, the need for supplementation or replacement nutrition, or significant difficulties with psychosocial functioning [6,52]. The DSM-5 identifies three main mechanisms that may lead to the development of ARFID: selective avoidance of many foods due to their sensory characteristics (so-called ‘picky eating’), reduced appetite or little interest in food, and fear of unpleasant consequences after a meal, such as choking, vomiting, abdominal pain, or bloating [6,52]. The prevalence of ARFID in the global adult population is estimated to range widely from approximately 0.3% to 3.1%, with these values varying depending on the study group and the diagnostic methods used [54]. Systematic analyses of studies on ARFID indicate a wide range of its prevalence—from 1.5% to as much as 64% in groups of patients with clinically diagnosed eating disorders. However, it is worth emphasizing that most of these studies are based on small samples, primarily including children and adolescents [6,55]. Furthermore, this disorder leads to insufficient nutrition or energy supply and is not associated with fear of weight gain, body image disturbances, or striving for a slim body shape [56].

### 3.3. Orthorexia Nervosa and Differential Diagnosis

Orthorexia is also a relatively new concept; it has not yet been officially classified as an eating disorder—it is not listed in either the ICD-11 or the DSM-V, and neither the American Psychiatric Association nor the World Health Organization recognize it as a distinct mental entity. Orthorexia nervosa (ON) is a proposed and evolving construct characterized by a pathological preoccupation with ‘healthy’ eating and rigid dietary rules. Individuals with orthorexia tendencies voluntarily implement numerous dietary restrictions, focusing on the process of preparing meals and controlling the origin of products [7]. Orthorexic symptomatology has been associated with a compulsive need to protect health and well-being through rigorous dietary practices [8,57], and even a small deviation from the adopted rules can cause a strong sense of guilt, shame or anxiety, which in turn may lead to increasingly restrictive dietary restrictions [7,58,59]. Some experts view orthorexia as a variant of ARFID, while others suggest that orthorexia is closer to obsessive–compulsive disorder, which influences inappropriate eating patterns [7]. The key distinction between ARFID and orthorexia lies in the type of anxiety accompanying the eating process: ARFID is associated with fear of the immediate, short-term consequences of food intake, such as choking, sensory discomfort, or loss of appetite [5,60], while orthorexia is associated with anxiety about the potential long-term health effects of consumed products, e.g., the risk of chronic diseases [60,61]. No universally accepted diagnostic criteria for orthorexia nervosa have been established, and available assessment instruments, including ORTO-15, should be interpreted as measures of orthorexic tendencies or symptomatology rather than as diagnostic instruments [62,63].

### 3.4. The Intersection of Eating Disorders and Gastrointestinal Diseases

Individuals with a history of ED have been shown to be more likely to develop gastrointestinal conditions during their lifetime. This association was comparable between genders and applied to anorexia nervosa (AN), bulimia nervosa (BN), and binge eating disorder (BED) [12,64]. Patients with ED have an increased risk of overall mortality as well as an increased risk of suicide compared to the general population [2,9]. Eating disorders and internalizing disorders have been shown to independently correlate with gastrointestinal diseases. Research suggests a common genetic component for these conditions, and identifying common molecular pathways may provide a basis for developing therapeutic interventions for these conditions [12].

Available observational evidence supports potentially bidirectional associations between eating disorders and gastrointestinal diseases (Figure 1). EDs are frequently associated with GI pathology through observed links with dysbiosis and dysmotility, while GI conditions commonly co-occur with or may exacerbate disordered eating behaviors via symptom-specific anxiety or restrictive therapeutic diets. Observational studies support associations between celiac disease and AN, as well as an increased incidence of eating disorders among children with IBD [65]. A two-sample Mendelian randomization study reported evidence compatible with a potential causal effect of genetic liability to IBD on AN; however, such inference depends on the validity of instrumental-variable assumptions and should not be interpreted as experimentally established causality [66].

## 4. Eating Disorders and Disordered Eating in Inflammatory Bowel Disease (IBD)

Inflammatory bowel disease (IBD) is a group of conditions that commonly affects young adults. Disease manifestations (abdominal pain, bloody diarrhea, unintentional weight loss) depend on the disease location, subtype, and disease activity. The course is usually relapsing with periods of exacerbations and remissions [67,68,69]. Ulcerative colitis (UC) and Crohn’s disease (CD) are the primary disease entities within IBD. These conditions significantly reduce quality of life, contributing to limitations in daily functioning, difficulties in social and intimate relationships, and deterioration of mental and physical health [18,70,71]. IBD affects patients’ activity, social life, psychological well-being, and interpersonal relationships. The disease is also associated with lower health-related quality of life (HRQoL) [72,73].

Individuals with inflammatory bowel disease (IBD)—including Crohn’s disease (CD) and ulcerative colitis (UC)—are particularly susceptible to behavioral problems, including various forms of eating disorders (EDs) such as anorexia nervosa (AN), bulimia nervosa (BN), and binge eating disorder (BED) [74]. In a study of IBD patients, 4% were underweight, 24% were overweight, and 4% were obese. One-third of participants reported concerns about their body image, and over 10% reported disordered eating behaviors. It has been suggested that adolescents with IBD may experience disturbed eating patterns related to body image and weight [75]. Individuals with IBD also have a significantly higher incidence of new episodes of post-traumatic stress disorder, eating disorders, self-harm, sleep disorders, depression, anxiety disorders, and new mental health issues in general. The highest risk of developing additional psychiatric conditions was noted in boys aged 12–17 years and in patients with Crohn’s disease [76]. Additionally, analyses have shown that individuals with IBD who experience comorbid mental health conditions generate significantly higher healthcare costs compared to IBD patients without such comorbidities [76,77]. Mental health assessment remains insufficiently integrated into routine care for IBD patients. Further research is needed to develop the most effective methods for monitoring and providing psychological support to these patients [76].

Proper diagnosis of comorbidities associated with IBD is crucial, their scale is unknown, but they can interfere with treatment of the underlying disease, increase disease activity, and are associated with reduced coping skills, reduced quality of life, and impact psychological well-being [78]. A study was conducted in Ontario among 33,961 patients with Crohn’s disease and 32,389 patients with ulcerative colitis. The incidence rates of eating disorders were significantly higher in patients diagnosed with UC or CD than in the control group. The strongest point estimates were observed for ulcerative colitis cases in individuals aged ≤18 years. Children with immunological gastrointestinal disorders had more than twice the incidence of eating disorders compared to their peers without these conditions. In adults, this association was less pronounced, but still noticeable [79]. Additionally, one of the single-center prospective longitudinal studies conducted among children diagnosed with IBD, focusing on dietary patterns at the Children’s Clinical Hospital “Dr. Victor Gomoiu” in Bucharest, the capital of Romania, showed that a higher percentage of IBD patients were already (before diagnosis) exposed to an unhealthy diet, and the overall adherence to a healthy diet was reduced even before the diagnosis of gastrointestinal pathologies in children [80].

### 4.1. The Impact of Diet

Diet remains closely linked to IBD. IBD patients whose eating behaviors are unsupervised increase their risk of developing restrictive eating behaviors, consequently increasing the risk of malnutrition and nutritional deficiencies. The role of diet in the pathogenesis of IBD is intensively studied, and recent data suggest that the consumption of pro-inflammatory foods (such as red meat and refined grain products) may influence the composition of the gut microbiota and modulate the severity of inflammation. Eating patterns in IBD result from a complex interaction between inflammation, altered gut–brain communication, gut peptides, disease symptoms, cognitive, and psychological factors. Most patients obtain information about their diet from the Internet. Nutritional factors may constitute a bridge between the environment and the development of IBD, and appropriately selected dietary interventions are an integral part of the treatment of these diseases. Diet is likely to contribute to IBD pathogenesis, although the available evidence regarding specific dietary components is limited and indirect [6,81,82,83,84,85].

Variations in disease rates across the world and the increasing prevalence of IBD in regions adopting Western dietary patterns suggest a significant role for diet in this process. Potential mechanisms of dietary action include, among others, its influence on the permeability of the mucosal barrier and its impact on components of the immune system. Diet significantly shapes the composition and functioning of the gut microbiome and its metabolites, which, along with genetic and immunological factors, contribute to the pathogenesis of IBD [86,87]. Early detection of malnutrition, achieved through screening tools for nutritional status, is crucial for effective therapeutic management [88].

It has been demonstrated that after a diagnosis of Crohn’s disease (CD), patients experience significant changes in their habits and lifestyle: they limit their consumption of alcohol, drugs, and smoking. They also change their diet, eliminating foods high in sugars and fats. Higher levels of depression were also noted in these patients [89]. Multivariate analysis demonstrated that both the presence of Crohn’s disease and prior intestinal surgery were independently associated with an increased risk of micronutrient deficiencies [90]. A study conducted among 20 patients diagnosed with IBD from four hospitals in China demonstrated that most patients made dietary decisions based on their current disease status and dietary guidelines. Their decisions were also influenced by their past experiences. Although most patients changed their diet after diagnosis, these patients showed significant deficiencies in nutritional education, which resulted in these changes often being inconsistent with current dietary recommendations [91]. A prospective survey of 400 adults with IBD in the UK found that 57% of respondents believed that their diet influenced the course of their disease and could provoke flare-ups, and two-thirds of them gave up their favorite foods in an attempt to alleviate symptoms [92]. Based on data analysis from a study conducted in Poland, two dietary patterns (DP) were identified: “Processed high fat/sugar/salt/meat/dairy/potatoes” and “Semi-vegetarian.” High adherence to the “Processed” pattern was associated with the highest severity of gastrointestinal symptoms, while adherence to the “Semi-vegetarian” pattern was associated with the lowest severity of these symptoms. The study results indicate a significant, adverse effect of a diet based on ultra-processed foods on the frequency of gastrointestinal disorders in adults, while emphasizing the importance of healthy dietary choices [93].

### 4.2. Malnutrition in IBD Patients

Both ED and IBD primarily affect young people [10,94], and their symptoms may be similar. The fact that laboratory abnormalities related to malnutrition are often observed in both diseases can make it difficult to determine their coexistence. Malnutrition is observed in up to 70% of patients with active IBD and in approximately 38% of patients in remission. This condition correlates with an increased risk of disease exacerbations, increased hospitalization rates, increased likelihood of postoperative complications, and increased need for surgical treatment [88]. In one retrospective cohort study of 165 pediatric patients with IBD, over half of the patients (53.5%) were malnourished at diagnosis, a percentage that decreased to 46.9% during follow-up. At the same time, a significant increase in the prevalence of obesity in adulthood was observed compared to childhood (2.3% vs. 20.5%, *p* < 0.001) [95].

Malnutrition associated with IBD has multifactorial causes, but limited food intake plays a significant role, often resulting from avoiding food during disease flares to alleviate symptoms or from eliminating specific food groups from the diet [96]. Avoidance of certain food products during disease flares and higher disease activity have been shown to be associated with a high risk of malnutrition [97].

Food-related quality of life (FR-QoL) refers to the psychosocial consequences of food consumption, dietary habits, and the processes of eating and drinking, which may be significantly impaired in patients with inflammatory bowel disease. The FR-QoL-29 questionnaire is a reliable and valid tool, demonstrating close correlations with disease activity and the degree of resulting disability. In a study conducted in a population of IBD patients, the FR-QoL-29 questionnaire assessed the relationships between the scale scores and selected clinical parameters. Lower FR-QoL-29 values were found to correlate with higher serum albumin levels, younger age, greater inflammatory disease activity, and higher levels of IBD-related disability. Female patients with a stenotic phenotype, diagnosed with Crohn’s disease, and after IBD surgery demonstrated significantly poorer nutritional quality of life and require special clinical support [98]. In another study, a poorer FRQoL was demonstrated in patients with reduced appetite and restrictive eating behaviors associated with fear of negative consequences of eating, while a higher FRQoL was observed in those who had undergone prior surgery and with lower disease activity [85].

### 4.3. Risk Factors for ED in IBD

Early identification and appropriate management of eating disorders may facilitate improved mental and physical health outcomes in patients with IBD [74]. In one study, the percentage of adolescents with IBD at risk of developing ED was 9.6%. A direct correlation between the number of IBD relapses and the risk of developing ED has been demonstrated [99]. Many studies have focused on identifying risk factors for ED development among patients with IBD, which could contribute to paying special attention and increasing clinical caution in such patients.

Crohn’s disease [95], female gender [74,100], history of weight loss [95], history of previous eating disorder diagnosis, number of IBD-related surgeries, dissatisfaction with current body weight [74], longer disease duration [75,100], gastrointestinal symptoms occurring particularly frequently during disease flares, increased anxiety [75], coexisting skin symptoms, perianal lesions, and prednisolone use [95] have been shown to increase the risk of nutritional disturbances in patients with IBD. Genetic predisposition to anorexia nervosa (AN) has also been shown to be nominally correlated with an increased risk of acute gastritis and Crohn’s disease [101]. One study, however, found that the presence of AN was associated with a later risk of IBD, but the reverse was not demonstrated [3]. In one study, 77% of patients with IBD met the study-specific ORTO-15 threshold for orthorexic tendencies; because ORTO-15 does not constitute a diagnostic instrument for orthorexia nervosa, this finding should be interpreted as a measure of orthorexic symptomatology rather than diagnostic prevalence [102]. A longer time since diagnosis, older age at follow-up, the presence of diarrhea, and higher height and BMI at presentation were associated with a lower risk of poor nutritional status [95].

Beyond disease-specific somatic factors, specific psychological characteristics play a pivotal role in the pathogenesis of EDs within the IBD population. Disrupted interoceptive awareness—the reduced ability to accurately perceive and interpret visceral signals—is a well-established core feature of EDs. In patients with IBD, interoception may be further distorted by chronic abdominal pain and altered gastrointestinal motility, potentially mediating the onset of disordered eating [103]. Furthermore, alexithymia, characterized by difficulties in identifying and describing feelings, is highly prevalent in both IBD and Eds [104]. In the context of chronic gastrointestinal distress, alexithymia severely impairs emotion regulation, which can lead patients to adopt restrictive or binge-eating behaviors as a maladaptive coping mechanism [105]. Additionally, traits associated with borderline personality disorder (BPD), notably profound emotional dysregulation and impulsivity, have been linked to a higher risk of binge–purge behaviors. The presence of BPD traits not only increases susceptibility to EDs but also significantly complicates the clinical course of IBD through treatment non-adherence and amplified psychological distress [106]. Consequently, evaluating these psychological vulnerabilities—interoceptive deficits, alexithymia, and BPD traits—is crucial for fully understanding the IBD-ED overlap.

## 5. ARFID in Gastroenterology—New Diagnostic Challenges

In clinical practice, patients with ARFID referred to gastroenterologists often describe a very restricted diet, limited to a few “safe” foods, typically consumed in small amounts. They often prefer to eat at home to avoid comments or judgment from others, and the process of reintroducing foods can be associated with significant anxiety. Although eliminating trigger foods may alleviate symptoms, it usually does not eliminate them completely, forcing patients into a stressful trial-and-error process to identify their triggers. Up to 90% of IBD patients deliberately avoid certain foods to reduce abdominal pain or diarrhea during flare-ups. Specifically, as many as 92% avoid specific foods during active symptoms, and 74% continue this avoidance even in remission, often due to the fear that certain foods may trigger a relapse. Grossberg (2025) [22] found that 16.3% of patients with inactive IBD screen positive for ARFID. GI-specific anxiety was identified as the sole significant predictor of a positive ARFID screen in this population. Each one-point increase on the Visceral Sensitivity Index (VSI) was associated with a 3.3% increase in the odds of screening positive for ARFID. These findings suggest that food restriction in remission is driven by psychological processes and fear of symptoms rather than active inflammation. While such dietary modifications can improve quality of life, excessive elimination poses severe health risks. It is crucial to determine whether the extent of dietary restriction is a necessary symptom-management strategy or indicates a pathological behavior requiring intervention [6,22,24,79,99,107,108].

Recent cross-sectional studies using the Nine-Item ARFID Screen (NIAS) have reported substantial proportions of adults with gastrointestinal disorders screening positive for ARFID-related symptoms or risk, ranging from approximately 10.2% to more than 53%, depending on the population, instrument, and disease characteristics [6,18,19,20,21,24]. These estimates should not be interpreted as diagnostic prevalence because NIAS-based screening may identify adaptive, symptom-driven food avoidance in patients with gastrointestinal disease.

### 5.1. Eosinophilic Esophagitis and ARFID

Eosinophilic esophagitis (EoE) is associated with an increased risk of developing avoidant/restrictive food intake disorder (ARFID) in both pediatric and adult populations [27,28]. A pediatric study of children with confirmed EoE reported that approximately 37% screened positive for ARFID using pediatric screening instruments, compared with 3% of matched controls. Screen-positive participants had lower quality of life and lower BMI z-scores compared with participants without a positive ARFID screen. This risk is notably elevated in children managed with food elimination therapy alone (70%) and those with multiple IgE-mediated food allergies; importantly, ARFID diagnosis in this group is not significantly related to histological disease activity or symptom severity [28]. In a retrospective tertiary-centre case series of adults with EoE referred for dysphagia or food impaction, approximately 4.5% met DSM-5 clinical criteria for ARFID. Because the cohort represented a selected referral population rather than an unselected EoE population, this estimate should not be interpreted as population-level prevalence [27]. The primary driver for food restriction is the fear of aversive consequences, specifically dysphagia and food impaction, which may stem from previous traumatic eating experiences [25,27].

### 5.2. Functional Dyspepsia/Gastroparesis/Upper Gastrointestinal Tract

Avoidant/restrictive food intake disorder (ARFID)-related screening positivity is frequent in functional and upper gastrointestinal motility disorders. In adults with gastroparesis, 77% screened positive on the NIAS, representing the highest reported proportion of ARFID screening positivity among the included gastrointestinal cohorts, with the most frequent phenotypes being appetite-related disturbances (84.2%) and fear of aversive consequences (75.6%) [29]. In pediatric cohorts, the proportion screening positive for ARFID ranged from 48.5% to 63.6% in gastroparesis and from 65.2% to 66.7% in functional dyspepsia (FD), depending on the screening instrument used. Notably, this risk does not correlate with objective physiological parameters, such as the degree of delayed gastric emptying or impaired fundic accommodation [30]. Population-based studies on disorders of gut–brain interaction (DGBI) indicate that the risk of ARFID is significantly higher among these patients compared to those without DGBI (34.6% vs. 19.4%), with the likelihood increasing alongside the number of affected GI anatomic regions. Among the DGBI group, the most common motivation is a lack of interest in eating (21.5%) [23]. Among the organic upper GI disorders evaluated in one cohort, achalasia showed the highest proportion of suspected ARFID based on screening (78.4%); this finding should be interpreted as screening positivity rather than confirmed diagnostic prevalence. However, experts highlight a significant methodological limitation: screening tools such as the NIAS may overestimate prevalence in organic diseases because they cannot distinguish between adaptive food avoidance driven by physical symptoms and the pathological avoidance characteristic of ARFID [24].

A summary of recent studies evaluating the prevalence and clinical characteristics of ARFID in GI patients is presented in Table 1.

The clinical implications of overlapping ARFID and GI disorders are profound. Patients screening positive for ARFID are at a significantly higher risk of severe malnutrition [6] and exhibit higher levels of high-sensitivity *C*-reactive protein (hsCRP), indicating elevated systemic inflammation [109]. Furthermore, avoidant eating behaviors strongly correlate with increased severity of depressive symptoms, anxiety, and poorer health-related quality of life [24]. Interestingly, while active disease symptoms and inflammatory parameters remain significantly associated with ARFID risk, studies have demonstrated no significant association between ARFID risk and demographic factors such as age, sex, or race/ethnicity [6].

However, researchers caution that the frequency of positive NIAS results may be overestimated in these populations, particularly among patients with active disease [21,24]. This highlights the pressing need to refine ARFID assessment methods for patients with complex gastrointestinal disorders to avoid false-positive diagnoses [24]. Despite this, clinical recognition remains a major challenge. In a small Mayo Clinic pilot study, unaided clinician recognition of eating disorder cases identified by formal screening was poor, with reported sensitivity of 0% and specificity of 96.5% [18]. Given the single-centre pilot design, small sample size, and absence of a formal ARFID diagnostic interview, this finding should be interpreted as evidence of potential under-recognition rather than as sufficient evidence to mandate universal screening across gastroenterological settings.

## 6. Functional Bowel Disorders and Eating Disorder Symptoms

Disorders of gut–brain interaction (DGBIs), formerly referred to as functional gastrointestinal disorders (FGIDs), include conditions associated with, among others, visceral hypersensitivity and abnormal motility of various segments of the gastrointestinal tract [110]. DGBIs constitute a group of clinical entities characterized by chronic or recurrent gastrointestinal symptoms that cannot be explained by a detectable organic cause. Since there are no definitive biomarkers or characteristic endoscopic findings, diagnosis and classification rely primarily on patient-reported symptoms [111,112]. Individuals with DGBI may experience numerous complaints, such as early satiety, postprandial fullness, bloating, nausea, vomiting, or epigastric pain [111,112,113]. Available data indicate an elevated risk of co-occurring eating disorders in individuals with DGBI [114,115].

In one study, it was found that a substantial percentage of patients diagnosed with eating disorders presented with at least one DGBI (88.2–95.5%), and between 34.8% and 48.7% reported the presence of three or more such conditions [115]. The most frequently observed group of DGBIs was functional bowel disorders, with irritable bowel syndrome (IBS) being the predominant entity, with a prevalence ranging from 43.9% to 58.8%. All analyzed types of eating disorders exhibited a significant positive correlation with the majority of DGBI categories. Individuals reporting more severe eating disorder symptoms were characterized by a higher mean number of DGBIs (3.03–3.34) compared to patients presenting with milder symptom severity (1.60–1.84) [115]. It has been investigated that among pediatric patients with eating disorders diagnosed with AN, the majority report gastrointestinal symptoms; these specifically include functional constipation (61%), functional dyspepsia (54%), and irritable bowel syndrome (25%), while 9% of patients meet the criteria for rumination syndrome [116]. Additionally, an analysis of gastrointestinal disorders in patients with eating disorders revealed that among those diagnosed with an eating disorder, 74.2% received their ED diagnosis prior to the diagnosis of their gastrointestinal disorder. The most common gastroenterological diagnoses in the study cohort were functional disorders, particularly among individuals with previously diagnosed eating disorders [14].

Irritable bowel syndrome (IBS) is among the most frequently diagnosed disorders of DGBIs, occurring in approximately 11% of the global population [114,117]; its prevalence is 1.5 to 3 times higher in women than in men [114,118]. This condition is chronic in nature and manifests with abdominal pain and altered bowel habits, which significantly reduces the quality of life of patients [114,117]. It has been demonstrated that irritable bowel syndrome (IBS) is associated with the occurrence of mood and sleep disorders, as well as anxiety [119].

Data suggest that eating disorders may promote the development of IBS in the long term, although the number of studies analyzing causal relationships between these conditions remains limited [60,120]. In patients with gastrointestinal diseases, the co-occurrence of eating disorders correlates with exacerbated psychiatric symptoms, including increased levels of stress and anxiety [60,121]. Concurrently, concerns arise that the use of restrictive dietary interventions in the treatment of IBS may additionally predispose individuals to the development of abnormal eating behaviors [60].

A cross-sectional study was conducted including 308 patients with irritable bowel syndrome (IBS) and 341 individuals with self-reported gluten-related disorders [122]. The applied self-report questionnaire was used to assess gastrointestinal symptom-specific anxiety, trait anxiety, negative affectivity, subjective severity of gastrointestinal symptoms, as well as quality of life and general well-being. In the IBS group, visceral anxiety was moderately to strongly associated with trait anxiety and IBS-specific quality of life, whereas in individuals with gluten-related disorders, it correlated with negative affectivity, the frequency of gastrointestinal symptoms, and well-being levels. Additionally, patients with the diarrhea-predominant IBS subtype (IBS-D) and the mixed subtype (IBS-M) obtained significantly higher Visceral Sensitivity Index scores compared to those with unclassified IBS (IBS-U) [122]. Another study conducted among individuals with irritable bowel syndrome demonstrated that the greater the severity of IBS symptoms, the higher the probability of co-occurring eating disorders and the lower the level of eating competence. In the analyzed group, 27% of individuals were classified as having a probable or highly probable eating disorder, and 29.1% reported current or past problems of this type. The analysis revealed a positive correlation between IBS symptom severity and scores on the 26-item Eating Attitudes Test (EAT-26), as well as a significantly lower Satter Eating Competence Inventory (ecSI 2.0™) score in participants with severe disease compared to those with a moderate course of the disease [114].

Studies indicate a significant association between irritable bowel syndrome (IBS) and specific eating disorders [50,60]. Among women with newly diagnosed, untreated AN, the prevalence of irritable bowel syndrome was estimated at 52.6% [50]. In IBS patients identified with eating disorder traits (according to the “sick, control, one-stone, fat, food”—SCOFF questionnaire), a significantly greater severity of orthorexia symptoms and greater disease symptom severity (IBS-SSS) were noted. This group was also characterized by higher levels of stress and anxiety, as well as a significantly lower food-related quality of life (FR-QoL) [60]. This is confirmed by analyses demonstrating a positive correlation between the severity of functional gastrointestinal symptoms and the propensity for orthorexia and emotional eating. It has been shown that the relationship between somatic symptoms and orthorexic tendencies is partially mediated by health anxiety. Conversely, the association between gastrointestinal complaints and emotional eating is explained by the exacerbation of orthorexia symptoms. This suggests that IBS patients, in an attempt to alleviate symptoms through rigorous dietary control, may develop orthorexic behaviors, which in turn can serve as a starting point for other forms of eating disorders, such as emotional eating, in which health anxiety plays a significant role [123].

## 7. Celiac Disease and the Risk of Eating Disorders

Gastrointestinal diseases classified as “organic” disorders encompass entities characterized by physiological changes within the digestive system, which frequently emerge in response to specific dietary components. In celiac disease, exposure to gluten leads to damage to the intestinal villi, manifesting as diarrhea, bloating, nausea, vomiting, and a general sense of fatigue, among other symptoms [24].

Celiac disease is a chronic, lifelong [124] systemic immune-mediated disorder triggered by exposure to gluten, leading to damage to the small intestinal mucosa, which results in both gastrointestinal symptoms and extraintestinal manifestations [125]. Along with the increased availability of diagnostic tools, a clear rise in the frequency of its diagnosis is observed worldwide [124,126,127,128], and the burden on patients as well as the costs associated with their treatment are constantly increasing [124]. The primary form of therapy remains a strict gluten-free diet, which allows for the resolution of symptoms and reduces the risk of long-term complications [129]. At the same time, both the chronic symptomatology and the necessity of lifelong adherence to restrictive dietary recommendations significantly burden the patients’ quality of life, although some data indicate that a longer time elapsed since diagnosis promotes better adaptation to the disease [128].

The strict elimination of gluten requires significant control over food choices, which may lead to an excessive focus on eating, dietary habits, or body weight. In some patients, this promotes the development of abnormal eating patterns, including binge-eating episodes, compensatory behaviors, or extreme dietary restrictions characteristic of eating disorders [126,130,131]. Shared pathophysiological elements have also been noted—including links between the immunoregulatory mechanisms of celiac disease and metabolic pathways associated with diabetes or anorexia nervosa—which may predispose individuals to the co-occurrence of these diseases [126,132]. Extensive population-based analyses indicate the existence of a bidirectional relationship between immune-mediated conditions and various forms of eating disorders [79,133]. Additionally, the population of patients with celiac disease exhibits a demographic profile similar to that of individuals with eating disorders, including a predominance of women, young individuals, and those of Caucasian descent [126].

A study conducted in Ontario investigated the association between celiac disease and the occurrence of eating disorders. The study was conducted among 14,718 patients with celiac disease. An increased incidence of eating disorders was observed in both pediatric and adult populations following the diagnosis of immune-mediated gastrointestinal diseases. It was demonstrated that the risk of developing eating disorders was particularly high in pediatric patients [79].

Conversely, in another survey study conducted among adult patients with celiac disease, participants completed a validated eating disorder questionnaire (Eating Attitudes Test-26). In individuals suffering from celiac disease, the presence of depressive symptoms, difficulties in adapting to chronic living with the disease, and body weight dissatisfaction were factors significantly increasing susceptibility to the development of eating disorders [126]. In a study of women with celiac disease, 71% met the study-specific ORTO-15 threshold for orthorexic tendencies; given the absence of standardized diagnostic criteria for orthorexia nervosa, this estimate should be interpreted as screening-defined orthorexic symptomatology rather than diagnostic prevalence. A positive correlation was demonstrated between the age of the subjects and the scores obtained in the ORTO-15 test. Individuals with orthorexic tendencies prepared their meals independently significantly more often (94%) compared to participants without such tendencies (78%). Simultaneously, individuals with elevated orthorexic tendencies paid less attention to the caloric value of consumed products (46% vs. 69%). In 64% of individuals with orthorexic tendencies, compared to 8% in the control group, thoughts about food elicited heightened anxiety [134].

## 8. Microbiota Alterations in Eating Disorders

Immune system dysregulation and microbiome alterations have been observed in patients with ED. Inflammation and microbiome changes have been recognized as essential features of IBD; however, microbiome alterations are associated with numerous diseases [135]. A detailed summary of microbiome alterations and their parallels in gastrointestinal diseases is presented in Table 2. A significant relationship has been demonstrated between the composition of the gut microbiome and the occurrence of mental disorders such as depression, anxiety, and eating disorders [136]. It has been shown that individuals diagnosed with mental disorders (MD) exhibit an increased abundance of bacteria from the *Parabacteroides* genus. Thus, it has been demonstrated that *Parabacteroides* may act as a potential mediator or biomarker of mental disorders, although its impact likely depends on the specific species and requires further research. The utility of microbiome analyses in studies on disorders with a complex genetic-environmental etiology has been confirmed [136]. The observed differences can only be partially attributed to nutritional status, encompassing both malnutrition and obesity. Consequently, it is hypothesized that the gut microbiome plays a key role in this complex mechanism; nevertheless, further research is needed regarding the specificity of dysbiosis and its significance in the development of eating disorders [35]. Crucially, direct head-to-head microbial comparative studies directly contrasting patient cohorts with primary eating disorders against cohorts with IBD or IBS within the same protocol are currently lacking in the literature. Consequently, the conceptualized ‘shared dysbiosis signature’—characterized predominantly by the depletion of short-chain fatty acid-producing *Firmicutes* and the expansion of pro-inflammatory *Proteobacteria*—represents an inference derived from parallel observational studies rather than co-recruited comparative trials. Caution is required when inferring shared pathophysiological mechanisms from these separate observational datasets.

### 8.1. Microbiota Alterations in AN

An important mediating element in the gut–brain axis is the inflammatory response. Significant correlations have been demonstrated between immunological parameters and the composition of the gut microbiota, involving several bacterial genera [137]. Microbiome causality in AN is currently based on mouse FMT models rather than human RCTs [31,32]. Human evidence consists mainly of case reports and observational data, precluding conclusions on whether dysbiosis is a cause or result of starvation [33]. To this end, the WFSBP consensus underscores that while overall microbial alpha-diversity remains largely preserved, distinct shifts in community structure—specifically the depletion of butyrate-producing species and the expansion of mucin-degrading taxa—coincide with clinical gastrointestinal dysmotility, including delayed gastric emptying and chronic constipation. In murine FMT models, transplantation of microbiota from individuals with AN has been associated with altered weight recovery and changes in hypothalamic and adipose-tissue gene expression [51]. These findings provide biological plausibility for a role of the microbiota in AN, but they do not establish a causal effect in humans. Well-designed controlled human studies are required to determine whether microbiome alterations play a causal role in AN and whether microbiome-targeted interventions have clinically meaningful therapeutic effects [41]. Additionally, the current imbalance in the presentation of specific eating disorders reflects a genuine evidence gap; while the AN microbiome has been synthesized across multiple cohorts [32,33], research regarding BN and ARFID is currently in its infancy [36,41]. Sparse data regarding pediatric ARFID indicate reduced species richness and a dysbiotic signature characterized by the enrichment of *Enterobacteriaceae* and *Bacteroidetes* (notably *B. vulgatus*), alongside a depletion of *Bifidobacterium* [40,41].

It has been shown that specific inflammatory mediators may participate in the underlying mechanisms of selected mental disorders, including anorexia nervosa (AN) [35,137]. In particular, an association between elevated *Interleukin-18* levels and anorexia has been demonstrated, suggesting the involvement of inflammatory processes in the pathophysiology of this disorder. A genetic predisposition to a higher proportion of bacteria from the *Clostridiaceae1* family and the *Butyrococcus* genus is associated with a lower risk of anorexia, whereas the presence of the *Parabacteroides* genus correlates with an increased probability of its development. It was also found that selected cytokines and chemokines, including *Interleukin-18* may slightly increase the risk of anorexia [35].

Changes in cytokine concentrations during the hospitalization of patients with AN were inversely associated with alterations in the abundance of selected bacteria from the *Lachnospiraceae* family and the *Dialister* genus. Changes in *IL-1β* levels correlated negatively with certain representatives of *Lachnospiraceae*, and positively with the *Bacteroides* genus. Conversely, in the long term, positive correlations were observed between changes in *TNF-α* and *IL-15* concentrations and variations in the abundance of bacteria with potential metabolic and anti-inflammatory significance, such as *Anaerostipes* and *Faecalibacterium*. There are close links between the inflammatory response and the composition of the gut microbiota in anorexia nervosa, and microbiome alterations may co-participate in modulating the immunological processes accompanying eating disorders [137].

A study by Andreani NA et al. evaluated changes in the gut microbiome composition in hospitalized female adolescents with anorexia nervosa (AN) during treatment and at a one-year follow-up. It was demonstrated that the microbiome composition at admission was significantly associated with disease duration and previous weight loss, while changes occurring during therapy correlated with energy intake, weight gain, and normalization of hormonal parameters. The greatest deviations in the microbiota structure compared to healthy individuals were observed during the acute starvation phase and at low body weight; these differences gradually diminished with weight gain, especially one year after treatment initiation [33]. Slightly different observations were made in a study by Morisaki, Y. et al., which showed that despite gradual weight gain and improvement in psychological functioning, individuals with AN maintained a distinctly different, abnormal gut microbiota profile compared to age-matched healthy women. During treatment, an increase in the detection frequency of bacteria from the *Lactiplantibacillus* genus was observed, and an increase in the abundance of *Bifidobacterium* during hospitalization was significantly associated with greater weight gain at the one-year follow-up [138]. In another study among patients with AN, it was shown that elevated levels of *Clostridium* clusters I, XI, and XVIII, and reduced levels of *Roseburia* spp. did not change even after weight gain or diet normalization [139]. Conclusions from these observations indicate that weight normalization does not automatically lead to the restoration of a normal gut microbiota composition in patients with AN [138,139]. It was shown that the gut microbiota at the time of admission had prognostic value, including regarding the risk of rehospitalization and long-term treatment outcomes [33].

In female patients with AN, regardless of the clinical subtype, reduced intra-individual bacterial diversity was also found compared to healthy subjects [140]. In a cross-sectional study that evaluated the gut microbiota composition in 30 patients with AN compared to an equal-sized control group, patients with anorexia nervosa exhibited an increased proportion of bacteria from the *Lachnospiraceae* family and the *Eubacterium hallii* genus, alongside a concurrent decrease in the abundance of *Ruminococcaceae*, *Faecalibacterium*, and *Subdoligranulum*. The analysis confirmed that taxa belonging to the *Lachnospirales* order and the *Lachnospiraceae* family were characteristically overrepresented in the AN group [34]. Additionally, another cross-sectional study on a group of 16 female patients diagnosed with AN compared to 14 female control patients demonstrated that the microbiome of the study group was characterized by an increased abundance of *Akkermansia muciniphila* (a bacterium responsible for regulating the immune response, strengthening the intestinal barrier, and preventing the development of metabolic syndrome), while the microbiome of the control group was dominated by *Roseburia* (a bacterium belonging to the *Firmicutes* phylum), *Agathobacter*, and *Faecalibacterium* [141]. Significant correlations between microbiome composition and clinical parameters were also demonstrated. Lower body weight and BMI were associated with a higher abundance of bacteria from the *Bacteroidota* phylum and *Bacteroides* genus, while a higher BMI correlated positively with the proportion of *Firmicutes* and *Subdoligranulum* [34].

Importantly, the obtained results indicate the existence of different types of intestinal dysbiosis depending on the clinical subtype of anorexia nervosa. Researchers Monteleone AM et al. compared the microbiological and metabolomic profiles of women with the restricting subtype of anorexia nervosa (ANR), the binge-eating/purging subtype (ANBP), and healthy controls. In AN patients, regardless of the clinical subtype, reduced bacterial diversity was found compared to healthy individuals. When compared to the ANR group, ANBP patients were characterized by a significantly higher relative abundance of bacteria from the *Bifidobacterium* genus, *Bifidobacteriaceae* and *Eubacteriaceae* families, and the *Bifidobacteriales* order. Simultaneously, a reduced abundance of bacteria belonging to the *Odoribacter* and *Haemophilus* genera, as well as the *Pasteurellaceae* family and *Pasteurellales* order, was observed in this group. This suggests differences in the interactions between gut microorganisms and metabolites, which may be significant for understanding the gut–brain axis in eating disorders [140].

Analyses of microbiota composition in the pathogenesis of AN indicate significant disturbances within numerous bacterial taxa, including the *Clostridium* genus, which showed associations with the severity of abnormal eating behaviors and mental health parameters [31]. This is confirmed by the results of The MICROBIAN study, where a significant positive correlation was found between the severity of anxiety and depression symptoms and bacteria from the *Firmicutes* phylum [142]. Concurrently, another study demonstrated that the severity of depressive symptoms was inversely associated with the abundance of *Faecalibacterium* [34].

In AN patients, an enrichment of bacterial functional modules involved in the metabolism and degradation of neurotransmitters has been demonstrated, and selected bacterial structural variants correlate with metabolic features characteristic of this disorder. Analyses indicated that bacterial metabolites present in the serum may participate in signaling pathways linking altered gut microbiota with metabolic and behavioral phenotypes observed in AN; however, causal mediation has not been established. Additionally, significant alterations within the gut virome were observed in the subjects, including a reduction in viral–bacterial interactions [31].

The potential involvement of the microbiota in the pathogenesis of AN is supported by animal model studies utilizing fecal microbiota transplantation. In one study, transplanting microbiota from AN patients into germ-free mice maintained on an energy-restricted diet resulted in reduced weight gain in the animals and changes in gene expression in the hypothalamus and adipose tissue, which was associated with disturbances in energy metabolism and eating behaviors. The obtained preclinical results suggest that intestinal dysbiosis may represent an associated contributing factor in the pathophysiology of anorexia nervosa, though human cross-sectional data cannot currently differentiate whether dysbiosis is a cause or a secondary consequence of severe starvation [31]. However, it should be noted that the results are not unequivocal—another study conducted on rats did not show that microbiota transplantation contributed to additional weight reduction compared to the control group [143].

Interventions aimed at microbiota modulation yield promising results. In a study by Agustí A et al., rats with an eating disorder characterized by a lack of control over food intake were administered *Bacteroides uniformis CECT 7771* (*B. uniformis*), resulting in decreased anxiety and mitigation of binge-eating episodes, which was associated with an effect on the brain’s reward response [39]. In a retrospective single-centre study of 100 children with AN, selected differences in intestinal microbiota, gut–brain-related biochemical measures, reported clinical efficacy, and 6-month recurrence rates were observed in patients receiving probiotic supplementation in addition to standard treatment compared with controls [144]. Because treatment allocation was non-randomized, the sample size was limited, and the study was conducted at a single centre, these findings should be considered hypothesis-generating rather than evidence of an established therapeutic effect. The study does not establish that probiotic supplementation improves gut–brain axis function or long-term clinical outcomes. Controlled randomized studies are required before probiotics can be recommended as an established treatment for AN. Despite everything, this underscores the potential significance of therapeutic strategies such as an appropriately modified diet or the use of live-microorganism-based biotherapeutics [138].

### 8.2. Microbiota Alterations in Bulimia

Bulimia is associated with reduced levels of pro-inflammatory cytokines. In the case of bulimia, a positive association with the *Lonchococcus* genus and an inverse relationship with the presence of *Eubacterium hallii* have been demonstrated. Pro- and anti-inflammatory factors, such as *VEGF-A* and *TNF*, exhibited weak but significant associations with the occurrence of this eating disorder [35].

### 8.3. Microbiota Alterations in Binge Eating Disorder (BED)

Binge Eating Disorder (BED) is associated with a downward trend in alpha diversity and the expansion of potentially pro-inflammatory taxa such as *Enterobacteriaceae* [38]. In contrast to AN, BED is characterized by a depletion of *Akkermansia muciniphila*. In animal models, administration of selected microbiome-targeted interventions, including *Bacteroides uniformis*, *Bifidobacterium*, and *Faecalibacterium prausnitzii*, has been associated with reduced binge-like behavior and anxiety-related phenotypes. These preclinical findings provide biological plausibility but cannot be directly extrapolated to therapeutic efficacy in humans [31,36,39].

### 8.4. Microbiota Alterations in ARFID

It is also worth noting that a different profile of microbiota alterations is observed in the case of avoidant/restrictive food intake disorder (ARFID). In a study by Ye Q et al., a relative enrichment of taxa belonging to the *Enterobacterales* order and *Enterobacteriaceae* family, as well as the *Bacteroidaceae* family and *Bacteroides* genus, was found in children with ARFID, whereas bacteria from the *Actinobacteriota* phylum, including the *Bifidobacteriales* order and the *Bifidobacterium* genus, were more frequently observed in healthy children. The differences primarily concerned taxonomic composition, while overall microbiota diversity indices did not show significant deviations between the groups. Functional analyses did not reveal significant differences in major metabolic pathways; however, an enrichment of selected enzymes related to carbohydrate metabolism and an increased presence of antibiotic resistance genes, particularly to macrolides, were observed in children with ARFID. In one pediatric cohort, ARFID was associated with differences in selected gut microbial taxa and functional features. These findings are consistent with a potential association between ARFID and the gut microbiome; however, their causal significance remains uncertain and requires replication in larger longitudinal studies [40].

## 9. Limitations and Future Research Directions

### 9.1. Limitations

The clinical and pathophysiological integration of eating disorders (EDs) and gastroenterological pathology presented in this review must be evaluated within the context of several methodological constraints inherent to both the source literature and the design of this article.

First, a primary methodological vulnerability in current clinical literature is the widespread reliance on brief, self-report psychometric screening instruments—such as the Nine-Item ARFID Screen (NIAS), the EAT-26, or the SCOFF questionnaire—rather than structured, clinician-administered diagnostic interviews. These self-report modalities are subject to important diagnostic confounding and may not reliably differentiate between adaptive, symptom-congruent nutritional modifications (e.g., the clinically indicated avoidance of dietary triggers during active inflammatory bowel disease flares or to facilitate mucosal healing in celiac disease) and the maladaptive, psychopathology-driven restrictive eating patterns that define ARFID. Consequently, the exceptionally elevated ARFID-related screening-positivity rates documented in patients with gastrointestinal dysmotility and functional disorders—ranging from 66.7% in functional dyspepsia to 77% in gastroparesis—likely represent inflated statistical upper bounds rather than true psychiatric comorbidity rates, underscoring the need for structured clinical validation.

Second, a critical empirical gap exists due to the paucity of prospective, head-to-head clinical trials utilizing co-recruited cohorts of GI and ED patients. The majority of hypothesized shared biological, immunological, and microbiomic pathways are extrapolated from parallel observations of separate clinical cohorts, rather than direct, concurrent comparisons under identical experimental protocols. Third, a significant research asymmetry persists within the current metagenomic literature; while the gut microbiota in anorexia nervosa (AN) has been synthesized across multiple well-characterized cohorts, the profiling of the microbiome in bulimia nervosa (BN), binge eating disorder (BED), and ARFID remains in its nascent stages and is characterized by a scarcity of high-powered human trials. Furthermore, evidence establishing a causal role for the microbiome in ED pathophysiology is heavily reliant on murine fecal microbiota transplantation (FMT) models. Parallel human data remain overwhelmingly cross-sectional, which precludes definitive conclusions regarding whether observed taxonomic shifts represent primary etiological drivers or secondary physiological sequelae of chronic starvational stress and altered gastrointestinal transit.

Finally, regarding the methodology of the present review, its narrative framework—though systematically structured in compliance with SANRA guidelines and utilizing thorough database queries—is inherently susceptible to selection bias. Unlike a registered systematic review and meta-analysis, this study does not feature a pre-specified, rigid protocol or quantitative statistical pooling of heterogeneous datasets.

### 9.2. Overall Strength and Hierarchy of Evidence

The evidence base evaluated in this review is heterogeneous and should therefore be interpreted according to study design and diagnostic validity. Population-based longitudinal studies and studies using formal clinical or structured diagnostic assessments provide greater inferential strength than small cross-sectional or self-report studies. Prospective and multicentre observational studies using validated screening instruments provide evidence regarding associations and screening positivity but do not establish clinical diagnostic prevalence or causality. Single-centre cross-sectional studies and convenience samples provide more limited generalizability. Case series and case reports are primarily hypothesis-generating. Animal and other preclinical studies are useful for exploring biological plausibility but cannot establish causality or therapeutic efficacy in humans. Genetic instrumental-variable approaches, including Mendelian randomization, may provide evidence compatible with causal relationships when their assumptions are satisfied, but these estimates remain dependent on instrument validity and are not equivalent to experimentally demonstrated causality. Overall, substantial heterogeneity in populations, diagnostic instruments, thresholds, study designs, and outcomes precludes direct pooling of estimates and limits the validity of a single summary prevalence across gastrointestinal disorders.

### 9.3. Future Research Directions: Bridging Gastroenterological and Systemic Biological Perspectives

To advance the clinical management of comorbid eating and gastrointestinal pathologies, future research must shift from isolated, localized metagenomic profiling to an integrated, system-level framework. In alignment with the World Federation of Societies of Biological Psychiatry (WFSBP) consensus statement, restrictive eating pathologies should be conceptualized as complex metabo-psychiatric conditions [51]. Interdisciplinary clinical trials must prioritize elucidating how the gut dysbiosis, altered short-chain fatty acid (SCFA) profiles, and compromised mucosal barrier permeability detailed in this review interact with systemic biological vulnerability.

Specifically, longitudinal investigation should target the diagnostic and therapeutic validation of biomarkers that bridge the enteric and central nervous systems. Future research should systematically evaluate how the gut microbiota and key regulatory peptides—specifically the adipokine leptin and the novel ghrelin antagonist LEAP-2—interact with central biological substrates. This includes monitoring blood-based biomarkers of neural plasticity (BDNF) and sensitive indicators of subclinical axonal or astroglial damage (neurofilament light chain [NfL], tau proteins, and glial fibrillary acidic protein [GFAP]) to determine if central functional connectivity abnormalities resolve during nutritional rehabilitation or persist as stable trait markers of relapse risk [51].

Additionally, exploring the genetic and epigenetic architecture of these comorbidities represents a crucial frontier. Investigating the DNA methylation of highly replicated candidate genes—such as *NR1H3* (involved in lipid and energy homeostasis) and *TNXB* (regulating connective tissue formation)—is highly indicated. Clinically, abnormal connective tissue integrity has been linked to both myocardial alterations and overlapping gastrointestinal complaints, such as severe dysmotility and low body mass index, suggesting a shared, systemic vulnerability pathway.

To address the clinical under-recognition of these comorbidities, major international gastroenterological societies (including the AGA, ECCO, BSG, ESPGHAN, and ESPEN) should collaborate to develop and prospectively evaluate evidence-based screening and case-finding strategies for eating disorders in gastroenterology clinics and emergency departments, rather than assuming that universal routine screening should be implemented. Furthermore, the deployment of passive digital biomarkers (such as actigraphy and smartphone-based Ecological Momentary Assessment) should be leveraged to track real-time behavioral and somatic fluctuations in naturalistic environments, bypassing self-report biases. Ultimately, utilizing machine learning algorithms to synthesize these complex, multimodal datasets—bridging gastroenterological, metagenomic, molecular, neuroimaging, and digital phenotypic signatures—will be essential to transition from empirical dietary management to highly personalized, target-specific therapeutic interventions [51].

## 10. Conclusions

The coexistence of eating disorders, particularly ARFID and orthorexic symptomatology, with gastrointestinal diseases, including inflammatory bowel disease (IBD) and irritable bowel syndrome (IBS), represents a clinically relevant and often underdiagnosed diagnostic challenge. Current evidence supports increased clinical vigilance and targeted assessment of disordered eating and nutritional risk, particularly in patients with substantial dietary restriction, persistent gastrointestinal symptoms, nutritional compromise, or psychological distress. Screening instruments may be useful for initial case finding, but a positive screening result should not be equated with a clinical diagnosis and should prompt further clinical assessment when appropriate. At present, the available evidence is insufficient to support universal routine screening of all patients attending gastroenterology services irrespective of disease activity or clinical risk. The complex, bidirectional relationship between the manifestation of gastrointestinal symptoms and eating psychopathology necessitates the application of an integrated, multidisciplinary model of care, combining the competencies of a gastroenterologist, psychiatrist, and dietitian. Systematic patient education, fostering the development of health-promoting dietary behaviors, should become an integral element of the therapeutic process. It is essential to conduct further well-designed prospective studies that will allow for a precise understanding of the gut–brain axis mechanisms and the development of standardized diagnostic and therapeutic algorithms in this patient population. However, these conclusions must be interpreted in light of both the existing literature and our methodological approach. The available evidence relies heavily on self-reported screening tools rather than structured clinical diagnostic interviews, and there remains a notable scarcity of prospective, head-to-head clinical trials. Furthermore, as a narrative review, this study synthesizes the literature without the quantitative, statistical pooling of data typical of systematic meta-analyses, which may introduce selection bias and limits the generalizability of the findings. Acknowledging these limitations places the current evidence in a more accurate context and underscores the critical need for rigorous, standardized future research.

## Figures and Tables

**Table 1:** Studies evaluating ARFID-related symptoms, screening positivity, and clinically diagnosed ARFID in adult and pediatric populations with gastrointestinal disorders. Studies are grouped by gastrointestinal population and listed chronologically within each group. Where 95% confidence intervals were not reported in the original publication, we have estimated them from reported sample size and proportion using the Wilson method; estimated intervals are preceded by “~”.

First Author/Year	Design	n, GI Population (Subtype)	Instrument and Cutoff	ARFID-Related Outcome (Screening Positivity or Clinical Diagnosis; 95% CI)	Key Covariates	Limitations	Conclusion
**Inflammatory bowel disease**							
Robelin 2021 [18]	Cross-sectional pilot; single-centre (Mayo Clinic, USA)	n = 98 adults with IBD	NIAS, cutoffs **Picky ≥ 10**, **Appetite ≥ 9**, **Fear ≥ 10**	**10.2%** NIAS+ (95% CI ~5.0–18.0)	Compared unaided physician judgement vs. NIAS—unaided clinician sensitivity for ED was **0%** (specificity 96.5%)	Small n; pilot design; single tertiary centre; no formal ARFID diagnostic interview	Demonstrated possible under-recognition of eating disorder symptoms without formal screening and supports further evaluation of screening approaches in IBD.
Yelencich 2022 [6]	Cross-sectional; single centre (UCLA IBD Center, USA)	n = 161 adults with IBD (45.3% CD, 51.6% UC, 3.1% IBD-U)	NIAS, **cutoff ≥ 24** (composite); PG-SGA for nutritional risk	**17.0%** NIAS+ (28/161; 95% CI 11.7–23.6)	ARFID+ strongly linked to malnutrition: **60.7%** vs. 15.8% (*p* < 0.001). Associated with active disease symptoms. **No association** with age, sex, race/ethnicity, or BMI.	Single-centre; convenience sampling; NIAS not formally validated in IBD; cross-sectional design precludes causal inference	First large IBD-ARFID study; Positive ARFID screening was associated with a higher prevalence of malnutrition; the cross-sectional design does not establish causality.
Yin 2023 [19]	Cross-sectional, multicentre (4 tertiary centres, China)	n = 372 hospitalised adults with IBD	NIAS (Chinese-version), conventional Picky/Appetite/Fear cutoffs; FLEQ-Ch for food literacy	**32.5%** NIAS+ (121/372; 95% CI 27.8–37.5)	Elevated risk associated with active CD and adherence to restrictive dietary regimens. Inverse correlation with **food-literacy score** (FLEQ-Ch).	Hospitalised inpatients (selection bias toward higher disease activity); generalisability outside China unclear; food-literacy data self-reported	Highest screening positivity reported among the included hospitalized IBD cohorts.
Tu 2025 [20]	Cross-sectional, multicentre (4 tertiary hospitals, China)	n = 483 adults with IBD	NIAS (Chinese-version)	**20.3%** NIAS+ (98/483; 95% CI ~16.8–24.0)	Highest risk in active CD and those on specific restrictive dietary regimens; dietary attitudes mediated risk.	Chinese-only cohort; instrument-translation effects; no concurrent biomarkers of nutritional status	Supports an association between active CD and higher ARFID-related screening positivity.
Burton-Murray 2024 [21]	Cross-sectional; single centre	n = 101 adults with UC in clinical remission	NIAS (standard cutoffs); EDE-Q8 for body-image-driven ED symptoms	**11.0%** NIAS+ (95% CI ~5.8–18.4); **30%** had other cognitive/behavioural ED symptoms on EDE-Q8	In remission, NIAS screening positivity was lower than in active disease cohorts, while other ED-related symptoms remained common.	Single centre; remission cohort only; NIAS does not capture shape/weight concerns	Highlights value of pairing NIAS with EDE-Q to capture full ED spectrum.
Grossberg 2025 [22]	Cross-sectional, multicentre (BIDMC and Loyola, USA)	n = 325 adults with IBD (49.5% CD, 45.5% UC)	PARDI-AR-Q (ARFID subscale)	**17.8%** PARDI-AR-Q+ (95% CI 13.7–22.5); active vs. inactive disease 51% vs. 40%, n.s.	ARFID+ patients were **younger**, had **shorter disease duration** and **worse psychosocial functioning** (*p* < 0.05).	Two urban academic centres; retrospective disease-activity data; PARDI-AR-Q not previously validated in IBD	First multicentre PARDI-AR-Q study; PARDI-AR-Q screening positivity was similar to estimates reported in other IBD cohorts.
**Irritable bowel syndrome and disorders of gut–brain interaction**							
Flack 2026 [23]	Population-based internet survey (UK + USA)	n = 4002 adults; 1704 (42.6%) with ≥1 Rome IV DGBI	NIAS (standard subscale cutoffs); Rome IV diagnostic questionnaire	**34.6%** NIAS+ in DGBI vs. **19.4%** in non-DGBI; **aOR 1.67** (95% CI 1.43–1.94)	Adjusted for age, sex, ethnicity, mood disorders. Dominant subscale: **lack of interest in eating** (21.5%), then sensory avoidance (18.1%), then fear of aversive consequences. Findings replicated in both countries.	Self-report data; NIAS may overestimate ARFID where GI-driven restriction is rational; doctor-diagnosed celiac and IBD excluded from DGBI category	Largest dataset to date; Supports a population-level association between DGBI and ARFID-related screening positivity.
Fink 2021 [24]	Cross-sectional, single centre (Northwestern, USA)	n = 289 adults with achalasia, celiac, EoE, or IBD	NIAS (composite cutoffs)	**53.7%** NIAS+ overall (achalasia **78.4%**, other organic GI 35–57%)	Higher NIAS scores in patients on a physician- or dietitian-prescribed diet; ARFID correlated with anxiety and depression.	Single centre; convenience sample; authors caution that NIAS likely *overestimates* ARFID in organic GI disease because it cannot distinguish necessary symptom-driven restriction from pathological avoidance	Cautionary benchmark for NIAS interpretation in organic GI disease.
Nicholas 2021 [25]	Cross-sectional, online survey (USA)	n = 2610 adults aged 18–44 self-identifying as picky eaters	Self-report ARFID symptom and GI-symptom items	Subgroup analyses (not a single prevalence figure)	Picky eaters with GI symptoms had significantly higher **fear-of-aversive-consequences** scores than those without GI symptoms.	Self-selected “picky-eater” sample (recruitment bias); no clinician-confirmed GI diagnoses; cross-sectional	Identifies fear-driven motivation as a shared ARFID–GI mechanism.
**Celiac disease**							
Bennett 2022 [26]	Retrospective survey; single centre (Vanderbilt, USA)	n = 137 adults with biopsy-confirmed celiac disease	ARFID-cl (modified NIAS-equivalent) self-report survey	**57%** suspected-ARFID (78/137; 95% CI 48.4–65.4)	No differences in age, sex, BMI, nutrient deficiencies, or bone disease vs. ARFID-negative. Food and social burden on the Impact-of-Gluten-free-Diet Questionnaire most predictive. Gluten-free-diet adherence and biopsy healing **not better** in ARFID+.	Retrospective; instrument is a modified survey rather than the published NIAS; selection bias toward symptomatic patients attending a tertiary clinic	Strikingly high prevalence; argues against the assumption that restriction is uniformly disease-driven.
**Eosinophilic esophagitis**							
Ketchem/Dellon 2022 [27]	Retrospective case series; tertiary centre (UNC, USA)	Adults with EoE referred for dysphagia or food impaction	Clinical ARFID criteria (DSM-5); retrospective chart review	First adult EoE–ARFID case series; prevalence not population-derived (denominator was incident EoE diagnoses, not all EoE patients)	All patients had **severe strictures** and high psychiatric-comorbidity burden; two-thirds on centrally acting medications.	Small retrospective series; selection bias; likely underestimates true prevalence (only documented cases captured)	Established adult EoE–ARFID as a distinct, clinically severe phenotype.
Murphy 2025 [28]	Cross-sectional; paediatric tertiary centre	Children with EoE	Validated paediatric ARFID screening (PARDI-AR-Q/Y-NIAS)	**37%** positive screen (vs. 3% in matched controls)	ARFID patients 10× more likely to report GI disorders and **5×** more likely to have elevated triglycerides and hs-CRP.	Single centre; paediatric cohort; screening instrument may inflate prevalence; cross-sectional	Demonstrates substantial overlap between EoE and ARFID-related screening positivity in a pediatric tertiary-care cohort.
**Gastroparesis and functional dyspepsia**							
Hollis/Burton Murray/Parkman 2024 [29]	Prospective; consecutive adult patients at academic centre (Temple, USA)	n = 107 adults with gastroparesis (4 h gastric retention 33.5 ± 21.8%)	NIAS (standard cutoffs); PAGI-SYM for GI severity	**77%** NIAS+ (82/107; 95% CI 67.9–84.5). Subscale positivity: appetite 84%, fear 76%, picky 45%.	Gastroparesis diagnosis **preceded** eating difficulties in 38%; eating difficulties preceded gastroparesis in 17%. Female predominance (84%).	Single centre; tertiary referral bias; NIAS may overestimate given that symptom-driven food avoidance is rational in delayed gastric emptying	Reports the highest observed NIAS screening positivity among the included GI cohorts; temporal self-report data suggest that gastroparesis diagnosis often preceded eating difficulties.
Kaul 2024 [30]	Prospective longitudinal; pediatric tertiary care (USA)	Children (10–17 y) with gastroparesis, functional dyspepsia, or healthy controls	NIAS and PARDI-AR-Q (concurrent administration); 2-month follow-up	**Gp: 48.5%** (NIAS)/**63.6%** (PARDI-AR-Q); **FD: 66.7%/65.2%**; HC: 15.3%/9.7% (*p* < 0.0001 across groups)	Positive ARFID screen was **not related** to delayed gastric retention or abnormal fundic accommodation. 71.9% (NIAS)/53.3% (PARDI-AR-Q) of baseline positives remained positive at 2 months.	Single centre; small subgroup numbers; instrument-discordance (NIAS vs. PARDI-AR-Q) demonstrates measurement uncertainty	Demonstrates high screening positivity for ARFID-related symptoms in pediatric gastroparesis and functional dyspepsia, with persistence of screening-positive status over 2 months.

**Abbreviations**: aOR, adjusted odds ratio; ARFID, avoidant/restrictive food intake disorder; ARFID-cl, ARFID checklist (modified NIAS-equivalent); BIDMC, Beth Israel Deaconess Medical Center; BMI, body mass index; CD, Crohn’s disease; CI, confidence interval; DGBI, disorder of gut–brain interaction; DSM-5, Diagnostic and Statistical Manual of Mental Disorders, 5th edition; ED, eating disorder; EDE-Q8, Eating Disorder Examination Questionnaire 8-item; EoE, eosinophilic esophagitis; FD, functional dyspepsia; FLEQ-Ch, Food Literacy Evaluation Questionnaire (Chinese); GI, gastrointestinal; Gp, gastroparesis; HC, healthy controls; hs-CRP, high-sensitivity *C*-reactive protein; IBD, inflammatory bowel disease; IBD-U, IBD unclassified; n.s., non-significant; NIAS, Nine-Item ARFID Screen; PAGI-SYM, Patient Assessment of Upper Gastrointestinal Symptoms; PARDI-AR-Q, Pica, ARFID, and Rumination Disorder Interview–ARFID Questionnaire; PG-SGA, Patient-Generated Subjective Global Assessment; UC, ulcerative colitis; UCLA, University of California Los Angeles; UNC, University of North Carolina; Y-NIAS, Youth NIAS. **Instrument cut-off conventions:** NIAS subscale cutoffs follow Robelin et al. (2021) [18]: Picky ≥ 10, Appetite ≥ 9, Fear of aversive consequences ≥ 10. A positive screen is defined when at least one subscale meets its cutoff. Yelencich (2022) [6] used the composite NIAS cutoff of ≥24. PARDI-AR-Q follows Stern/Bryant-Waugh thresholds for screen-positive status. **Interpretive note:** NIAS and similar screens cannot reliably distinguish necessary, symptom-driven dietary restriction (e.g., avoidance of trigger foods during an IBD flare or in EoE-related dysphagia) from clinically significant ARFID. Reported prevalences should therefore be regarded as upper bounds requiring confirmation by structured diagnostic interview (e.g., PARDI) and multidisciplinary assessment. The exceptionally high prevalences in gastroparesis (77%), functional dyspepsia (66.7%), and achalasia (78.4%) likely reflect this measurement limitation rather than ARFID prevalence per se.

**Table 2:** Microbiome alterations across eating disorders and their parallels in inflammatory bowel disease and irritable bowel syndrome. Reported microbiome alterations across eating disorders and gastrointestinal diseases show partial overlap, including changes in selected butyrate-producing taxa and Enterobacteriaceae/Proteobacteria. However, these findings are heterogeneous and are derived predominantly from separate observational cohorts; therefore, they should not be interpreted as evidence of a validated shared dysbiosis signature.

Condition	Category	Diversity	Increased Taxa	Decreased Taxa	SCFAs/Metabolites	Key References
**Eating disorders**						
**Anorexia nervosa**	Psychiatric/restrictive	Inconsistent for α-diversity; β-diversity altered in most studies	*Methanobrevibacter smithii* *Akkermansia muciniphila* *Lachnospiraceae* (some species) *Clostridium* clusters I, XI, XVIII *Eubacterium hallii*	*Faecalibacterium prausnitzii* *Roseburia inulinivorans* *Ruminococcaceae* *Subdoligranulum* *Bacteroidetes* phylum (low BMI)	↓ butyrate; ↑ branched-chain fatty acids; altered tryptophan metabolism	Zhao 2024 Fan 2023 Andreani 2024 Yuan 2022 [31,32,33,34]
**Bulimia nervosa**	Psychiatric/binge–purge	Limited data; α-diversity broadly preserved	*Lachnospiraceae* shifts *Lonchococcus* Inconsistent ratio of *Firmicutes: Bacteroidetes*	*Eubacterium hallii* Pro-inflammatory cytokines *VEGF-A*, *TNF* weakly associated *Faecalibacterium prausnitzii*	Sparse data; possible ↓ butyrate-producers; ClpB-like bacterial proteins implicated as anti-melanocortin mimics	Tempia Valenta 2025 Ma 2025 Carbone 2020 [35,36,37]
**Binge eating disorder**	Psychiatric/binge	Limited data; trend toward ↓ diversity	*Enterobacteriaceae* *Bacteroidetes* *Clostridium*	*Akkermansia muciniphila* *Faecalibacterium prausnitzii*	Animal models: ↓ SCFAs; *Bacteroides uniformis* supplementation reduces binge-like behaviour	Tempia Valenta 2025 Guo & Xiong (2024)·Agustí 2021 [36,38,39]
**ARFID**	Restrictive (sensory/fear-based)	Chao1 ↓ (richness); Shannon and Simpson indices ↑ (paediatric data)	*Enterobacterales*/*Enterobacteriaceae* *Bacteroidaceae*/*Bacteroides vulgatus* *Escherichia coli* *Streptococcus thermophilus*	*Actinobacteriota* phylum *Bifidobacteriales*/*Bifidobacterium* *Prevotella copri* *Ruminococcus gnavus*	Glycosyltransferase GT26 enriched; ↑ macrolide-resistance genes; SCFA data absent	Ye 2023 Schneider 2024 [40,41]
**Gastrointestinal disease parallels**						
**Inflammatory bowel disease (CD and UC)**	Immune-mediated inflammatory	↓ α- and β-diversity; more pronounced in Crohn’s disease	*Proteobacteria*/*Enterobacteriaceae* *Escherichia coli* (adherent-invasive) *Ruminococcus gnavus* (CD) *Streptococcaceae*, *Veillonellaceae*	*Faecalibacterium prausnitzii* *Roseburia* spp. *Lachnospiraceae*, *Ruminococcaceae* *Coprococcus*, *Eubacterium*	↓ butyrate, propionate, acetate; ↓ SCFA biosynthesis pathways; ↑ H_2_S	Cao 2014 (meta-analysis) Lloyd-Price 2019 [42,43]
**Irritable bowel syndrome**	Disorder of gut–brain interaction	↓ α-diversity in IBS-D and IBS-M; preserved or ↑ in IBS-C	*Methanobrevibacter smithii* (IBS-C) *Enterobacteriaceae* *Fusobacterium*, *Desulfovibrio* (IBS-D) *Veillonella*, *Ruminococcus*	*Roseburia intestinalis* *Bifidobacterium* *Methanobacteriales* (severe IBS) *Lachnobacterium*	↓ butyrate; ↑ H_2_S (IBS-D); ↑ methane (IBS-C)	Pozuelo 2015 Li 2025 Villanueva-Millan 2022 [44,45,46]
**Shared dysbiosis signature across eating disorders and DGBI/IBD**						
**Convergent depletions:** *Faecalibacterium prausnitzii*, *Roseburia* spp., *Bifidobacterium*, other butyrate-producing Firmicutes—observed across anorexia nervosa, ARFID, IBD, and IBS. **Convergent expansions:** *Enterobacteriaceae*/*Proteobacteria*—a hallmark of intestinal inflammation, present in IBD, IBS, ARFID, and BED. **Reported metabolic alterations:** These include reduced faecal short-chain fatty acids, including butyrate, in selected cohorts; whether these changes represent a shared pathophysiological mechanism remains uncertain. **Divergent signatures:** *Methanobrevibacter smithii* is enriched in both anorexia nervosa and IBS-C but depleted in active IBD; *Akkermansia muciniphila* is enriched in AN but depleted in BED.						

**Abbreviations:** AN, anorexia nervosa; ARFID, avoidant/restrictive food intake disorder; BED, binge eating disorder; CD, Crohn’s disease; DGBI, disorder of gut–brain interaction; ED, eating disorder; H_2_S, hydrogen sulfide; IBD, inflammatory bowel disease; IBS, irritable bowel syndrome; IBS-C/-D/-M, IBS with constipation/diarrhoea/mixed predominance; SCFA, short-chain fatty acid; UC, ulcerative colitis. Up and down arrows indicate increased and decreased relative abundance or concentration vs. healthy controls. **Notes on evidence quality:** AN microbiome data derive from approximately 14 cohort studies (n ≈ 476 patients) synthesised in Zhao 2024 [32] and Andreani 2024 [33]. BN and BED data are limited to fewer than 10 small studies, summarised in Tempia Valenta 2025 [36]. ARFID microbiome data derive principally from one paediatric cohort (Ye 2023 [40], n = 135) and the conceptual review of Schneider 2024 [41]. IBD signatures are based on the Cao 2014 [42] meta-analysis. IBS signatures are derived from cross-cohort meta-analyses (Li 2025) [45] and subtype-specific work (Villanueva-Millan 2022) [46]. **Methodological note: Direct head-to-head studies comparing ED and GI cohorts are lacking; the shared-signature inference is based on parallel observation rather than co-recruited samples**.

## Data Availability

No new data were created or analyzed in this study. Data sharing is not applicable to this article.

## Abbreviations

The following abbreviations are used in this manuscript:

AGA	American Gastroenterological Association
AI	artificial intelligence
AN	anorexia nervosa
ANBP	binge-eating/purging subtype of anorexia nervosa
ANR	restricting subtype of anorexia nervosa
aOR	adjusted odds ratio
ARFID	avoidant/restrictive food intake disorder
ARFID-cl	ARFID checklist (modified NIAS-equivalent)
BED	binge eating disorder
BIDMC	Beth Israel Deaconess Medical Center
BMI	body mass index
BN	bulimia nervosa
BSG	British Society of Gastroenterology
CD	Crohn’s disease
CI	confidence interval
CRP	*C*-reactive protein
DGBI	disorder of gut–brain interaction
DP	dietary pattern
DSM-5	Diagnostic and Statistical Manual of Mental Disorders, 5th edition
EAT-26	Eating Attitudes Test, 26-item
ECCO	European Crohn’s and Colitis Organisation
ecSI 2.0	Satter Eating Competence Inventory, version 2.0
ED	eating disorder
EDE-Q/EDE-Q8	Eating Disorder Examination Questionnaire/8-item version
EoE	eosinophilic esophagitis
ESPEN	European Society for Clinical Nutrition and Metabolism
ESPGHAN	European Society for Paediatric Gastroenterology, Hepatology and Nutrition
FD	functional dyspepsia
FGID	functional gastrointestinal disorder
FLEQ-Ch	Food Literacy Evaluation Questionnaire (Chinese version)
FMT	faecal microbiota transplantation
FR-QoL/FR-QoL-29	food-related quality of life/29-item questionnaire
GERD	gastro-oesophageal reflux disease
GI	gastrointestinal
Gp	gastroparesis
H_2_S	hydrogen sulfide
HC	healthy controls
HPA	hypothalamic–pituitary–adrenal
HRQoL	health-related quality of life
hs-CRP	high-sensitivity *C*-reactive protein
IBD	inflammatory bowel disease
IBD-U	inflammatory bowel disease, unclassified
IBS	irritable bowel syndrome
IBS-C	irritable bowel syndrome with constipation
IBS-D	irritable bowel syndrome with diarrhoea
IBS-M	irritable bowel syndrome with mixed bowel habits
IBS-SSS	Irritable Bowel Syndrome Symptom Severity Score
IBS-U	irritable bowel syndrome, unclassified
ICD-11	International Classification of Diseases, 11th revision
IgE	immunoglobulin E
IL	interleukin
MD	mental disorder
NIAS	Nine-Item ARFID Screen
n.s.	non-significant
ORTO-15	15-item orthorexia self-report questionnaire
PAGI-SYM	Patient Assessment of Upper Gastrointestinal Symptoms
PARDI/PARDI-AR-Q	Pica, ARFID, and Rumination Disorder Interview/ARFID Questionnaire
PG-SGA	Patient-Generated Subjective Global Assessment
RCT	randomised controlled trial
SANRA	Scale for the Assessment of Narrative Review Articles
SCFA	short-chain fatty acid
SCOFF	Sick, Control, One stone, Fat, Food (screening questionnaire)
SMA	superior mesenteric artery
TNF	tumour necrosis factor
UC	ulcerative colitis
UCLA	University of California, Los Angeles
UNC	University of North Carolina
VEGF-A	vascular endothelial growth factor A
VSI	Visceral Sensitivity Index
WHO	World Health Organization
Y-NIAS	Youth Nine-Item ARFID Screen
